# Dual Actions of Ketorolac in Metastatic Ovarian Cancer

**DOI:** 10.3390/cancers11081049

**Published:** 2019-07-24

**Authors:** Laurie G. Hudson, Linda S. Cook, Martha M. Grimes, Carolyn Y. Muller, Sarah F. Adams, Angela Wandinger-Ness

**Affiliations:** 1 Department of Pharmaceutical Sciences, University of New Mexico Health Sciences Center, Albuquerque, NM 87131, USA; 2 Department of Internal Medicine, Division of Epidemiology and Biostatistics, University of New Mexico Health Sciences Center, Albuquerque, NM 87131, USA; 3 Department of Obstetrics and Gynecology, Division of Gynecologic Oncology, University of New Mexico Health Sciences Center, Albuquerque, NM 87131, USA; 4 Department of Pathology, University of New Mexico Health Sciences Center, Albuquerque, NM 87131, USA

**Keywords:** ovarian cancer, peri-operative period, non-steroidal anti-inflammatory drug (NSAID), ketorolac, Rac1, Cdc42, therapeutic targets, metastasis

## Abstract

Cytoreductive surgery and chemotherapy are cornerstones of ovarian cancer treatment, yet disease recurrence remains a significant clinical issue. Surgery can release cancer cells into the circulation, suppress anti-tumor immunity, and induce inflammatory responses that support the growth of residual disease. Intervention within the peri-operative window is an under-explored opportunity to mitigate these consequences of surgery and influence the course of metastatic disease to improve patient outcomes. One drug associated with improved survival in cancer patients is ketorolac. Ketorolac is a chiral molecule administered as a 1:1 racemic mixture of the S- and R-enantiomers. The S-enantiomer is considered the active component for its FDA indication in pain management with selective activity against cyclooxygenase (COX) enzymes. The R-enantiomer has a previously unrecognized activity as an inhibitor of Rac1 (Ras-related C3 botulinum toxin substrate) and Cdc42 (cell division control protein 42) GTPases. Therefore, ketorolac differs from other non-steroidal anti-inflammatory drugs (NSAIDs) by functioning as two distinct pharmacologic entities due to the independent actions of each enantiomer. In this review, we summarize evidence supporting the benefits of ketorolac administration for ovarian cancer patients. We also discuss how simultaneous inhibition of these two distinct classes of targets, COX enzymes and Rac1/Cdc42, by S-ketorolac and R-ketorolac respectively, could each contribute to anti-cancer activity.

## 1. Introduction

From 1975–2014 in the US, 5-year relative survival for invasive ovarian cancer has increased from 33.6% to 46.8% [1]. Annual age-adjusted death rates have dropped from 9.84/100,000 to 6.74/100,000 paralleling a drop in incidence in the same time period [1], but this mortality drop is modest relative to the 5-year survival increase. These patterns highlight that 5-year survival has increased substantially, but annual mortality has only dropped modestly. Women are surviving longer due to improved treatment, but there is little improvement in overall survival in late-stage disease [2]. Ovarian cancer remains the leading cause of death from gynecologic malignancy, primarily due to the emergence of drug resistant disease following front-line surgery and systemic chemotherapy [3,4,5,6,7,8].

The mainstays of ovarian cancer treatment include a defined combination of surgery and chemotherapy. Front line systemic chemotherapy includes platinum compounds and taxanes, with doxorubicin, topotecan, and gemcitabine as options in recurrent disease [9]. Additional therapies including anti-angiogenics and poly (ADP ribose) polymerase (PARP) inhibitors have been added to treatment regimens with the goal of extending the disease-free interval [9,10]. More recently, immunotherapies are being evaluated in clinical trials. Benefits of PARP inhibitors are most successful in the minority of patients carrying germline BRCA1/2 mutations [11], but currently less so in the broader ovarian cancer patient population [10]. Immune therapies targeting checkpoint blockades yield mixed results in clinical trials and toxicity is a concern [10]. There are ongoing efforts to identify new actionable targets in ovarian cancer and expand the therapeutic repertoire for this disease [3,4,5,7,8,9,12]. 

A range of strategies needs to be considered to improve the outcomes for ovarian cancer patients. In recent years, the peri-operative period has received greater attention as a unique and largely overlooked opportunity for intervention in cancer treatment. Surgery modifies the tumor environment in ways that may promote tumor cell dissemination, survival, and expansion [13,14,15,16,17,18]. Research has identified candidate mechanisms and the potential for therapeutic interventions to allay the consequences of surgery on tumor recurrence [15,17,18]. Although retrospective studies provide support for greater attention on the peri-operative window, randomized-control clinical trials are needed to resolve the potential of peri-operative strategies to improve cancer patient outcomes. In subsequent sections, we discuss supporting evidence of the benefits of peri-operative ketorolac administration in cancer patients and underlying mechanisms that may account for these observations in ovarian cancer.

## 2. Cancer Surgery and Metastasis

### 2.1. Peritoneum is a Site for Residual Disease

Ovarian cancer, like many epithelial cancers, spreads by direct extension to adjacent organs. However, and in contrast with many other cancers, ovarian cancer also disseminates throughout the peritoneal (abdominal and pelvic) cavity [4,19,20]. Tumor cells that detach from the primary tumor are transported by the peritoneal fluid or the circulatory system throughout the peritoneal cavity, which then “seed” the intraperitoneal surfaces from the diaphragm to the distal colon. Peritoneal spread may be quite extensive and surgical removal of peritoneal implants can lead to significant tissue disruption. Such cytoreductive surgeries have been suggested to facilitate the metastatic process [16,17,18,21]. Proposed mechanisms include: (1) surgical stress that reduces cell-mediated immunity and a concurrent pro-inflammatory response, (2) physical effects through the dissemination of more tumor cells into the circulating peritoneal fluid or blood, and (3) wound healing responses, such as inflammation, angiogenesis, and proliferation, triggered by the tissue trauma of surgery [15,17,18]. The accumulating evidence suggests that the post-surgical period is a favorable environment for the growth of new and existing microscopic residual disease. 

### 2.2. Exploiting the Peri-Operative Period to Improve Long-Term Ovarian Cancer Outcomes

There is increasing evidence that many factors associated with surgical treatment of primary tumors modulate the tumor environment in a manner that can promote residual cancer survival and growth [15,17,18]. This critical time, the period just before surgery extending to several weeks following surgery, or the peri-operative period, is arguably a short time relative to the length of time for tumor growth and spread both before and after surgery. However, a number of studies have reported that drugs and physiological responses in this critical window can influence long-term outcomes such as recurrence and mortality. For example, inhalational anesthetics such as isoflurane or desflurane are associated with increased risk of death compared to propofol-based intravenous anesthesia based on a retrospective analysis of over 7,000 patients [18,22]. In contrast, peri-operative use of aspirin as an antithrombotic is associated with improved outcomes in patients with biliary, gastric, colorectal, or breast cancer [18,23]. Similarly, the beta-blocker propranolol decreased biomarkers of metastasis and modulated the immune environment in breast cancer [24,25]. Cyclooxygenase (COX) inhibitors have been largely studied for cancer chemoprevention [26,27,28,29], but new evidence suggests that women who used non-steroidal anti-inflammatory drugs (NSAIDs) after an ovarian cancer diagnosis had improved disease-specific survival compared with never-users [30,31]. These findings and others indicate that the anesthetic and analgesic type and approach used in and/or after surgery can influence cancer recurrence and metastasis [15,17,18,32]. 

### 2.3. Non-Steroidal Anti-Inflammatory Drugs (NSAIDs) and Cancer

The benefit of NSAIDs as general chemopreventive and anti-cancer agents remains controversial due to mixed epidemiologic evidence [33,34,35,36,37,38,39,40]. The most compelling evidence linking anti-inflammation with survival is found in colorectal cancer (CRC), where aspirin use is associated with increased CRC survival (e.g., [41,42]). Understanding the effects of NSAIDs in cancer is hampered by differences in findings based on tumor type, limited knowledge on response stratification based on specific NSAID use, and lack of information on potential enantiomer-dependent pharmacologic activities of certain NSAIDs. Enantiomeric pairs may differ greatly in biologic response with profound birth defects due to the S-enantiomer of thalidomide serving as a dramatic example [43,44,45]. While many NSAIDs are developed only as the S-enantiomer, some NSAIDs, such as ketorolac, are administered as a 1:1 racemic mix of the R- and S-enantiomers with the S-forms having inhibitory activity toward COX enzymes [46,47,48,49,50,51,52]. Although the R-forms have negligible to no activity against COXs [46,48,49,53,54,55,56], there is mounting evidence that R-enantiomers are distinct chemical entities and precedence for pharmacologic activities dictated by R-enantiomers of specific NSAIDs against novel (non-COX) targets [57,58,59,60,61,62,63,64].

## 3. Peri-Operative Use of Ketorolac

Ketorolac was approved by the US Food and Drug Administration in 1989 as the first injectable NSAID [65]. Originally marketed as Toradol^®^ (injection and tablet forms, Roche Laboratories, Nutley, New Jersey, USA), it is currently available as a generic drug. Ketorolac is non-narcotic but provides opioid-level pain management, thereby reducing narcotic requirements. In cancer, ketorolac may be used to control cancer-associated pain and is used as an analgesic during and after cancer surgeries [66,67,68,69,70,71,72]. 

### 3.1. Peri-Operative Ketorolac and Breast Cancer Survival

A 2010 breast cancer study [73] reported that among the 55% of women who received ketorolac, there was a decreased risk of breast cancer relapse (Hazard Ratio (HR) = 0.4, 95%CI = 0.1–0.8) with a particular reduction in relapses in the first 24 months of follow-up [74,75]. However, other analgesics (sufentanil, ketamine, and clonidine) did not confer this benefit [73]. Subsequent analyses included either ketorolac or diclofenac (another NSAID), and exposure to either one was combined in the analyses [76]. In one breast center (n = 172 patients), ketorolac/diclofenac was a strong predictor of recurrence-free survival (multivariate adjusted HR (aHR) = 0.2, 95%CI = 0.07–0.4) with recurrences reported in 6.9% of NSAID users at 60 months relative to 29.6% of non-users (*p*-value < 0.001). There were too few deaths for multivariate analyses, but mortality was 5.5% in users at 60 months relative to 20.7% in non-users (*p*-value < 0.001). In another breast center (n = 162 patients) ketorolac users had half the recurrences (3.0%) at 24 months versus non-users (6.6%), but the differences were not significant. There were fewer deaths among ketorolac users (3.4%) at 24 months than among non-users (7.9%), but not significantly so. More recently, ketorolac was associated with decreased distant recurrences in breast cancer patients (aHR = 0.59, 95%CI = 0.37, 0.96), perhaps driven by high-BMI patients (aHR = 0.55, 95%CI = 0.31, 0/96) [77]. While the evidence above suggests that ketorolac has an effect in clinical practice, the evidence is limited and is not specific to ovarian cancer. These studies also did not adjust for the propensity to receive ketorolac and had a limited ability to assess the more robust outcome of cancer-specific survival due to a short follow-up period.

### 3.2. Peri-Operative Ketorolac and Ovarian Cancer

We conducted an analysis of ovarian cancer survival as a function of peri-operative ketorolac administration [78]. Inclusion criteria for patients identified from the New Mexico Tumor Registry were: invasive, epithelial ovarian cancer, diagnosis age 40–79 years, diagnosis 2004–2006, and surgery at an Albuquerque hospital (only 3 hospitals provide this level of surgery). Diagnosis years were chosen so that each patient had at least 6 years follow-up through December 31, 2012. Medical records were abstracted for all analgesics and anesthesia used before hospital admission, during surgery and hospital stay, and given at discharge. Of 138 potential cases, eight had no surgery (palliative care only or died before surgery), one had surgery in another state, and medical records were not located for six, leaving 123 women in the final analysis. Peri-operative ketorolac was used in 14% of the women and was more likely to be received by younger women (versus older) (*p* < 0.05), other factors were not significantly different. At the 60-month follow-up, 3/17 ketorolac-treated (18%) and 40/92 non-treated patients (43%) had died of ovarian cancer (log-rank test *p*-value = 0.09). Stratified log-rank tests for categorical factors such as age group, American Joint Committee on Cancer (AJCC) stage, completion of chemotherapy as planned, and receipt of neoadjuvant chemotherapy showed a consistent ketorolac survival benefit in each stratum [78]. The survival benefit of ketorolac was also evident in the proportional hazards analysis when adjusted for age at diagnosis, AJCC stage, completion of chemotherapy as planned, and receipt of neoadjuvant chemotherapy. The adjusted HR for ovarian cancer–specific mortality associated with peri-operative ketorolac (yes versus no) was 0.30 (95% confidence interval (CI), 0.11–0.88). While these findings must be interpreted cautiously because they are only partially controlled for the propensity to receive ketorolac, they suggest that, similar to the breast cancer data, peri-operative ketorolac reduces ovarian cancer-specific mortality.

### 3.3. Peri-Operative Ketorolac and Other Cancers (Non-Ovarian)

In contrast to breast and ovarian cancer, in patients with prostate cancer there was no survival value for the intraoperative administration of ketorolac during prostatectomy [79,80]. In one study, no significant difference was found between general anesthesia plus post-operative ketorolac-morphine analgesia (n = 158) and general anesthesia plus intraoperative and post-operative thoracic epidural analgesia (n = 103) in biochemical recurrence-free survival, cancer-specific survival, or overall survival [80]. In another analysis of 1111 patients, the incidence of biochemical recurrence-free survival was compared in patients receiving epidural analgesia, intravenous ketorolac, sufentanil, clonidine, and ketamine in varying combinations. Sufentanil was associated with an increase in cancer relapse, but neither epidural analgesia nor other analgesics, including ketorolac, were associated with a statistically significant effect on biochemical recurrence-free survival (*p* > 0.05).

There are relatively few studies available for additional cancer types. However, the effects of ketorolac usage were studied in a group of non-small cell lung cancer (NSCLC) patients, and a group of kidney cancer patients who received major surgery [76]. Ketorolac administration was combined with diclofenac administration in some analyses, and the combined variable was referred to as “NSAID use”. Of the 227 kidney patients, only 13 received ketorolac and 6 received diclofenac and they were not analyzed further. For NSCLC, there was a decrease in distant metastases (adjusted HR = 0.16, 95%CI = 0.04, 0.63) and mortality (adjusted HR = 0.55, 95%CI = 0.31, 0.95) with NSAID administration. For ketorolac alone, there was a significant decrease in mortality risk (adjusted HR = 0.41, 95%CI = 0.23, 0.70, *p* < 0.001). A second study of 563 NSCLC patients [81] receiving an NSAID for postoperative pain management found that the majority of patients received ketorolac (67%). Although celecoxib or ibuprofen administration was not predictive of overall survival, patients receiving ketorolac displayed better overall survival (*p* = 0.05) without differences in recurrence-free survival [81]. There are no clear answers for the lack of benefit with ketorolac administration in prostate cancer patients compared to other cancers, but collectively, the evidence supports further studies to determine whether ketorolac administration in the peri-operative window has the potential to improve patient outcomes. 

## 4. Dual Pharmacologic Actions of Ketorolac 

In the retrospective studies on ketorolac usage and cancer outcomes discussed above, patients received ketorolac in the peri-operative period. This raises the question of how a short duration, non-cytotoxic treatment can lead to durable survival benefit in cancer patients. As reviewed by Horowitz et al. [17] and Hiller et al. [18], the peri-operative period may promote metastatic disease in patients by a number of mechanisms, including (1) peri-operative factors such as psychological stress and mechanical stress associated with surgery and tumor excision, (2) increases in neuroendocrine and paracrine factors such as catecholamines and prostaglandins, (3) modulation of the immune system, and (4) modulation of angiogenic and growth factors. Together, these mechanisms may enhance the survival, implantation, and growth of residual tumor cells either directly or indirectly by modifying the tumor microenvironment. The authors assert that this convergence of events occurring during the short peri-operative window are critical to long-term cancer outcomes and may offer opportunities for interventions during the peri-operative period to improve patient outcomes.

### 4.1. Cyclooxygenase (COX) Inhibition by S-ketorolac

#### 4.1.1. COX Enzymes in Ovarian Cancer 

Prostaglandins are necessary for normal ovarian functions, such as ovulation, and are produced within the ovary [82]. In ovarian cancer, inflammation and downstream inflammatory mediators are associated with initiation and progression. This is believed to be through contributions to genomic instability via DNA damage by reactive oxygen species elevated at sites of inflammation and regulation of antiapoptotic pathways by cytokines and growth factors [82]. COX-1 and 2 are overexpressed in ovarian tumors [83,84,85,86,87,88,89] and correlate with worse features, such as angiogenesis and proliferation. COX enzymes catalyze the conversion of arachidonic acid into prostaglandin H2, which is the precursor for all prostaglandins and thromboxane A2. Prostaglandins and thromboxane are lipid signaling molecules that can promote cell proliferation, angiogenesis, metastasis, and inhibit apoptosis. Both COX enzymes are important in ovarian cancer but may be differentially expressed in less aggressive Type 1 and more aggressive Type 2 tumors [82,88]. Elevated COX-1 mRNA expression is associated with Type 2 tumors [88,89] and Type 1 tumors are more likely to have high COX-2 mRNA levels [88]. Higher COX-1 mRNA expression was associated with shorter disease-free and overall survival, whereas COX-2 expression was associated with shorter disease-free survival amongst type 2 tumors [88]. Given the relationships between inflammation, aberrant COX expression and adverse consequences in ovarian cancer [82], COX inhibition by NSAIDs has been considered as a possible strategy to combat this disease.

NSAIDS are either COX-1 selective, COX-2 selective, or non-selective as inhibitors of COX enzymes. S-ketorolac is a potent and non-selective COX inhibitor with somewhat lower half-maximal inhibitory concentration (IC_50_) values for COX-1 than COX-2 in purified enzyme assays [63]. Ketorolac is significantly more potent than the non-selective COX inhibitors indomethacin or diclofenac in in vivo analyses of NSAID function for alleviation of pain and inflammation [53]. These functional outcomes are due to COX inhibition and attributed to the S-enantiomer as the R-enantiomer is more than 100 times less potent for inhibition of either COX enzyme [52,56,63,65]. Additional studies will be needed to discern whether COX selectivity of an NSAID is an important factor in cancer risk or tumor response. 

#### 4.1.2. COX Inhibitors and Ovarian Cancer Outcomes

The vast majority of published studies on NSAIDs and ovarian cancer outcomes are focused on the risk of developing ovarian cancer rather than peri-operative use. A number of reports conclude that NSAID use reduces ovarian cancer risk, but findings differ between studies [29,30,34,90,91,92,93,94,95,96,97]. Low dose aspirin appears to be protective [30,90,93,96,97] and a recent study suggested that long-term, high-quantity use of non-aspirin NSAIDs is associated with increased ovarian cancer risk, although the authors state that the finding requires confirmation [96]. Other studies find that any NSAID decreases risk [94], but there appears to be a greater advantage to aspirin use [93]. A large observational study of pooled individual data for 7694 women across multiple studies and self-reported regular use of NSAIDs before ovarian cancer diagnosis did not find an association with survival (disease-free or overall) [95]. However, the authors noted that when they restricted the analyses to the subset of studies with clear definition of NSAID use or non-use (non-use as less than once per week), a survival benefit was detected with use of any NSAIDs [95]. The authors also noted that improved and consistent definitions for NSAID use before and after diagnosis is needed [95] to help resolve the differences in reported study results. 

Randomized clinical trials of ketorolac in cancer have provided comparison in efficacy of pain relief rather than insights into patient survival outcomes. However, in one randomized clinical trial, the NSAID celecoxib was investigated by adding it to chemotherapy (docetaxel plus carboplatin) because elevated COX-2 protein expression is detected in ovarian cancer patients [85,86,87] and the transcription of COX-2 can be stimulated by taxanes (standard chemotherapy for ovarian cancer patients) [98,99]. Unfortunately, celecoxib with chemotherapy did not improve survival and 24% of women discontinued celecoxib due to side effects [99]. 

There is limited information on NSAID use after ovarian cancer diagnosis, but one recent study reported intriguing results. Reported NSAID use pre-diagnosis was not positively associated with ovarian cancer-specific survival. However, women who reported recent (current use in the past 2 years) use of aspirin or non-aspirin NSAIDs after diagnosis had improved ovarian cancer-specific survival compared with never-users [30]. This survival benefit was not found for post-diagnosis use of paracetamol (acetaminophen), suggesting that COX inhibitors may not be equivalent for survival outcomes. This study highlights the need for additional research on post-diagnosis use of NSAIDs and patient outcomes to supplement the studies on ovarian cancer risk. Although the mechanisms contributing to the observed benefits of NSAID use after diagnosis are not known, experimental studies suggest that tumor debris from chemotherapy stimulates proinflammatory cytokines and bioactive lipids, thereby stimulating tumor growth through numerous signaling pathways. A dual COX-2/soluble epoxide hydrolase inhibitor (PTUPB) markedly enhanced survival and delayed the onset of a debris-stimulated increase in inflammatory mediators in experimental models [100]. This finding suggests that multi-target strategies in the peri- or post-operative period may provide greater benefits than COX inhibition alone. 

### 4.2. Rac1 and Cdc42 Inhibition by R-ketorolac

#### 4.2.1. Rac1 and Cdc42 GTPases in Ovarian Cancer

Rac1 and Cdc42 are regulators of numerous functions related to tumor development, progression, metastasis, and chemo-resistance [101,102,103,104,105,106,107,108,109]. Rac1 activity and expression are frequently elevated in tumors and a recent meta-analysis of 1,793 patients in 14 studies concluded that Rac1 expression was a poor prognostic indicator across cancers [110]. Our analysis of the 298 Stage III and IV high-grade serous ovarian cancer (HGSOC) patients with outcomes data in The Cancer Genome Atlas (TCGA) demonstrate that high total RAC1 (but not CDC42) mRNA expression is associated with worse outcomes [105]. This finding concurs with another analysis of Rac1 as a prognostic factor in a cohort of 150 ovarian cancer patients [111]. 

#### 4.2.2. Identification of R-Ketorolac as a Rac1 and Cdc42 Inhibitor

Rac1 inhibitors have anti-tumor benefits in multiple cell and animal models of cancer, leading to vigorous efforts to identify clinically useful agents, reviewed in [108,109,112,113,114,115,116]. Based on a high-throughput screen of 8 Ras-related small GTPases against off patent, FDA-approved drugs and cheminformatics, we identified the R-enantiomer of ketorolac as a selective inhibitor of Rac1 and Cdc42 with no activity against the related GTPase RhoA [63]. The S-enantiomer was inactive against these GTPase targets [63,64] and R-ketorolac was inactive against COX enzymes. Although it has been long recognized that R-enantiomers of NSAIDs are poor inhibitors of cyclooxygenase activity [46,48,49,53,54,55,56], relatively little is known about potential pharmacologic activities or targets for these R-enantiomers. 

R-ketorolac is a noncompetitive inhibitor of Rac1 and Cdc42 with IC_50_ values of 0.57 and 1.07 μM, respectively [64]. Although a limited number of additional candidates were identified in the high-throughput screen, including R-naproxen, S-ibuprofen, and sulindac sulfide, more than 20 other NSAIDs are inactive against these proteins, confirming that the interaction with Rac1 and Cdc42 was not a general property of this class of drugs [63]. R-ketorolac inhibits serum and EGF-stimulated Rac1 and Cdc42 activation and downstream signaling through a proposed allosteric mechanism. Thus, identification of a novel activity for R-ketorolac has precedence in the literature and further indicates that R-enantiomers are distinct chemical entities compared to the corresponding S-forms [47,57,58,59,60,61,62]. 

#### 4.2.3. Experimental Evidence for Benefits of Rac1 Inhibition in Ovarian Cancer

Rac1 is a driver of numerous cancer-relevant phenotypes associated with worse patient outcomes. The broad impact of Rac1 on tumor cell behavior has led to consideration of Rac1 as a potential therapeutic target [102,108,112,113,114,115,116]. In ovarian cancer cell lines, knock down of Rac1 expression reversed epithelial to mesenchymal transition (EMT) [111,117], inhibited tumor cell migration and invasion [111], and reduced tumor growth in a xenograft model [111]. An inhibitor of Rac1 (NSC23766) decreased ovarian tumor cell migration, invasion, and matrix-metalloproteinase production [63,64,118].

**Figure 1:**
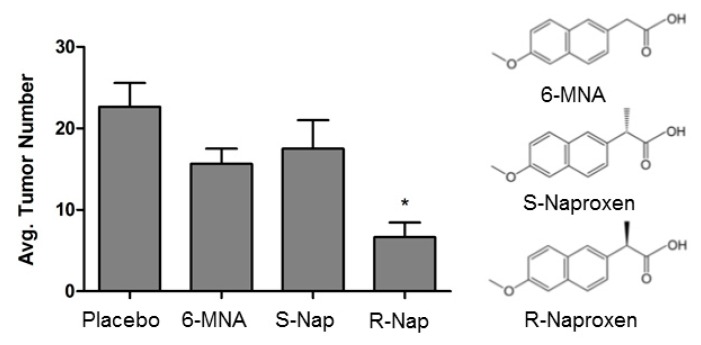
R-Naproxen reduces ovarian tumor peritoneal implants in a xenograft model. One day prior to intraperitoneal injection of green fluorescent protein (GFP)-tagged SKOV3ip cells into athymic nude mice, mice were administered placebo or the indicated treatments to approximate serum levels equivalent to human dosing. Drug was administered daily for 2 weeks. At this time, mice were sacrificed, and images of the peritoneal cavity were obtained. All tumors were counted and aided by GFP imaging. * *p* < −0.05 C = placebo; 6-MNA = 6-methoxy-2-naphthylacetic acid; S-Nap = S-naproxen; R-Nap = R-naproxen. 6-MNA is the active metabolite of the non-racemic NSAID nabumetone and is structurally similar to R-naproxen but lacks the alpha-methyl carboxylate (α-Me-COOH) determined to be essential for activity [63].

We find that R-ketorolac and R-naproxen inhibit tumor cell adhesion, migration, and invasion—all behaviors that are central to ovarian cancer metastasis [63,64]. R-ketorolac was tested using ovarian tumor cell lines and primary ovarian tumor cells isolated from patient ascites fluids [64]. R-ketorolac was an effective Rac1 inhibitor and decreased downstream signaling, as demonstrated by reduction of PAK1 and PAK2 phosphorylation [78]. R-ketorolac, but not S-ketorolac, inhibited Rac1-dependent cellular functions in ovarian cancer cell lines and primary cells including inhibition of growth factor-stimulated formation of filopodia, cell adhesion to fibronectin and type I collagen, development of invadopodia, and tumor cell migration [64]. The inhibitory effects of R-ketorolac and R-naproxen in cells are comparable to those of established Rac1 and Cdc42 selective inhibitors [64,119]. R-ketorolac [120] and R-naproxen (Figure 1) also inhibit ovarian tumor cell omental engraftment and tumor growth in vivo, with little impact of the S-enantiomers or an achiral metabolite, 6-methoxy-2-naphthylacetic acid (6MNA).

**Figure 2:**
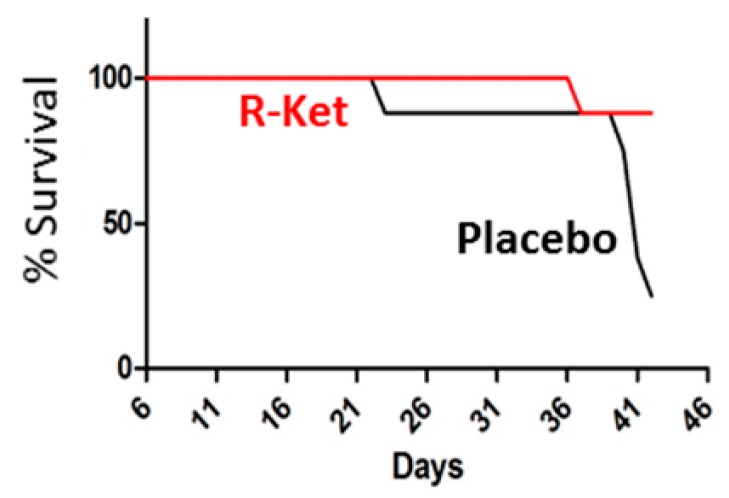
R-ketorolac improves survival in an in vivo xenograft model of ovarian cancer. OVCAR8 xenografts were established, then mice were treated with 10 mg/kg/day R-ketorolac or placebo by intraperitoneal injection for 6 weeks. The R-ketorolac group (R-Ket, red line) displayed improved survival compared to the placebo (black line) group (88% survival versus 25% survival). One mouse in the R-ketorolac group was lost at 37 days of treatment. For the placebo control group, mice were lost at day 23 (1), day 40 (1), day 41 (3), and day 42 (1, study termination). There was a statistically significant difference (*p*-value ≤ 0.0142) in survival between the placebo and R-ketorolac treatment groups, as determined using the Kaplan–Meier method and comparisons using the log-rank test. N = 8 mice/group.

Furthermore, R-ketorolac treatment led to increased survival of mice in a xenograft study using OVCAR8 ovarian cancer cells (Figure 2). These preclinical findings suggest that R-ketorolac may have beneficial actions in human ovarian cancer that could account for improved patient outcomes associated with peri-operative ketorolac use.

## 5. Is There Potential for Ketorolac in Ovarian Cancer Management? 

Racemic ketorolac is orally bioavailable and has been commercialized as topical (ocular, intranasal), injectable (intravenous or intramuscular), and oral formulations. This makes ketorolac an ideal candidate for human clinical trials. However, ketorolac has limitations as well. Ketorolac is contraindicated in patients with peptic ulcer disease, gastrointestinal bleeding, or advanced renal impairment, a common clinical issue in elderly women with ovarian cancer [65]. Use is restricted to a 5-day treatment course that could limit application for extended post-operative care in cancer patients. Within these limitations, we designed and conducted a “Phase 0” clinical trial. Ovarian cancer patients received racemic ketorolac for its FDA-approved indication in post-operative analgesia [78], then blood and peritoneal fluids were collected at intervals for 24 h. Ketorolac was distributed to the peritoneum within 1 hour after IV administration, and at 6 h, ketorolac levels in the peritoneal fluids were nearly equivalent to those present in the serum. At each of the time points (1, 6, and 24 h) after administration of the racemic drug, peritoneal fluids were enriched in R-ketorolac compared to the S-enantiomer. R-ketorolac achieved concentrations in the peritoneal fluids at or above the IC_50_ values for Rac1 and Cdc42 and was therefore predicted to inhibit these GTPase targets in cells obtained from this compartment. This prediction was supported by the finding of time-dependent inhibition of Rac1 and Cdc42 activity in cells retrieved from the peritoneal compartment of these post-surgical ovarian cancer patients after ketorolac administration [78]. Because R-ketorolac predominates in the peritoneal fluids and the S-enantiomer is virtually undetectable at 24 h, this indicates that the R-enantiomer is bioactive and accounts for the observed inhibition of the GTPases in vivo. 

**Figure 3:**
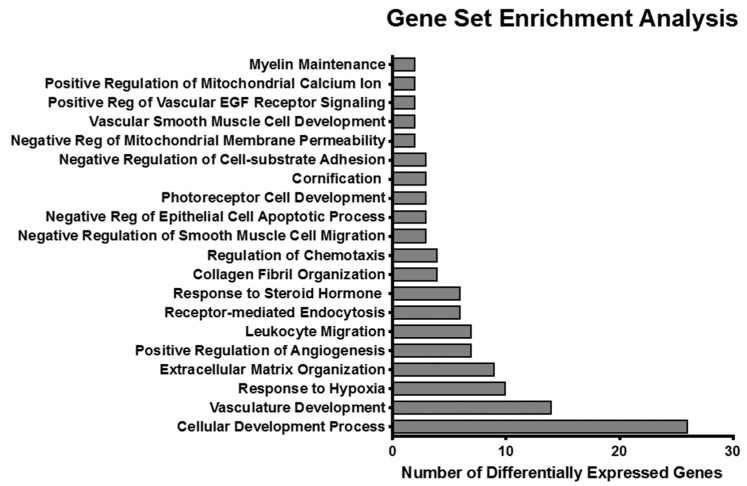
Ketorolac treatment down-regulates gene expression signatures in a mouse xenograft model of ovarian cancer. SKOV3ip xenografts were established, then mice were treated with racemic ketorolac at 1 mg/kg/day oral dosing or placebo for two weeks. Rac1 and Cdc42 activity was decreased by approximately 50% and 35% respectively, similar to what we found in clinical samples [78]. RNA-sequencing was conducted on tumors isolated from mice. Differentially expressed genes were identified using a threshold of > 2-fold change and a false discovery adjusted *p*-value cut-off of 0.05. All genes in this set were down-regulated by ketorolac.

To better understand the potential consequences of ketorolac treatment, we conducted gene expression analysis by RNA-sequencing of human ovarian cancer xenografts following ketorolac treatment. Mice were treated with a human equivalent dose of racemic ketorolac (1 mg/kg/day) for two weeks. A list of 53 significantly differentially expressed genes was generated from the RNA-sequencing analysis. Of 53 differentially expressed genes, 51 were downregulated by R-ketorolac. Enrichment analysis indicated that these genes affect several important biological functions. Using topGO to probe the Gene Ontologies (GO) database, we found the gene list to be enriched in 20 biological processes including 34 genes mainly involved in cellular development process, vasculature development, and response to hypoxia (Figure 3). We further analyzed the differentially significant genes by using Pathview [121,122] and DAVID [123,124] to probe the Kyoto Encyclopedia of Genes and Genomes (KEGG) to see if ketorolac had an effect on specific pathways. KEGG pathway analysis demonstrated that the down-regulated, differentially expressed genes were enriched in three significant pathways, including the HIF-1 signaling pathway (*p*-value 6.3^−06^), the PI3K-AKT signaling pathway (1.6^−02^), and the Focal Adhesion pathway (2.1^−02^). 

A limitation for applying findings from the mouse xenograft study to humans is based on the enantiomer-selective pharmacokinetics of ketorolac. There is greater retention of R-ketorolac compared to S-ketorolac after administration of racemic drug in mice and humans [56,78]. R-ketorolac is not significantly converted to S-ketorolac in mice or humans, but S-ketorolac is significantly converted to the R-enantiomer in mice, further favoring the ratio of R- to S-ketorolac [56]. Because humans do not convert S-ketorolac to R-ketorolac [56], the mouse studies reflect a greater proportion of R-ketorolac after administration of the racemic drug than might be true for humans after repeated doses. However, our findings support the conclusion that both the S- and the R-enantiomers of ketorolac are pharmacologically active in patients, and both the COX inhibitory, as well as the Rac1/Cdc42 inhibitory activities, should be taken into account when interpreting findings obtained after administration of the FDA-approved racemic drug.

## 6. Conclusions

The anti-cancer-relevant actions of R-ketorolac are evident in experimental models [63,64,120]. Based on the predominance of R-ketorolac over S-ketorolac in blood and peritoneal fluids, coupled with GTPase target inhibition after administration of racemic drug [78], we can infer that R-ketorolac is active as a Rac1/Cdc42 inhibitor in humans. Although Rac1/Cdc42 are recognized as attractive therapeutic targets in cancer, no selective inhibitors have advanced to human use. Our clinical study indicates that Rac1/Cdc42 inhibition can be achieved in humans at currently approved doses of the racemic drug. 

At this time, it is not known whether a subset of patients overexpressing Rac1 would be more likely to benefit from ketorolac. Indeed, elevated mRNA expression may not be the best determinant of tumor Rac1 activity for two major reasons. First, as an integrator of upstream signals generated by receptor tyrosine kinases, G-protein-coupled receptors, integrins, and other adhesion molecules, Rac1 activity varies based on factors within the tumor microenvironment [103,105,107]. Secondly, the activity of Rac1 is tightly controlled by a large network of modulatory proteins (e.g., Guanine nucleotide exchange factors, GEFs and GTPase activating proteins, GAPs) that have profound effects on net Rac1 activity and elevated activity would not be evident based on Rac1 mRNA or protein expression alone [103,105,107]. As one example, ARHGAP5 is a protein that stimulates GTP hydrolysis on Rac1 and the related family member RhoA (Ras homolog gene family, member A) resulting in decreased activation of these GTPases. The rs927062 variant of ARHGAP5 was found to be associated with increased endometrioid and invasive serious ovarian cancer risk [125] and a statistically significant decrease of ARHGAP5 protein which would be predicted to increase Rac1 and RhoA activity. It will be important to resolve the question of whether patients with elevated Rac1 expression and/or activity are most likely to benefit from ketorolac and if so, what markers would be appropriate for patient stratification.

Studies suggest that the anti-inflammatory actions of NSAIDs in ovarian cancer may confer benefits, but it is clear that additional research on the timing of NSAID use, whether pre-diagnosis, peri-operative, or post-diagnosis, is needed. Similarly, there is evidence that NSAIDs may not be equivalent in anti-cancer activity and more work will be required to discern the distinctions of specific NSAID use within the different timing contexts. 

A recent study tested pre-operative administration of ketorolac versus other NSAIDs in mouse models of cancer [126]. Ketorolac dosing before, but not after, resection of the subcutaneous tumors led to decreased metastatic recurrence and increased survival. Several other NSAIDs tested (indomethacin, aspirin, ibuprofen, diclofenac, celecoxib) were inactive or substantially less active than ketorolac for survival outcomes, although three highly selective COX-1 inhibitors displayed benefit similar in magnitude to ketorolac [126]. Modifications of ketorolac may improve efficacy compared to the parent drug. A 1,2,3-triazolyl ester of ketorolac called "15K" was synthesized and found to be substantially more potent than ketorolac for PAK and COX-2 inhibition, and cytotoxicity against lung and melanoma cancer cell lines [127]. In addition, this compound inhibited embryonic angiogenesis in a chorioallantoic membrane assay [128]. These and future experimental studies are critical for resolving key questions on the benefits of specific NSAIDs, including ketorolac or future derivatives, in cancer patients.

Taken together, our identification of R-ketorolac as a Rac1/Cdc42 GTPase inhibitor may help to explain the apparent benefits of racemic ketorolac in human breast and ovarian cancer patient survival, and why other NSAIDs have not yielded comparable findings. The current status of the literature indicates that both classes of targets (COXs and Rac1/Cdc42 GTPases) are important in ovarian cancer progression, metastasis, and patient outcome. Clinical trials using R-ketorolac alone would offer an opportunity to directly test the predicted benefit of a Rac1/Cdc42-selective inhibitor in ovarian cancer patients. R-ketorolac could circumvent current renal and hematologic toxicities that restrict the use of racemic ketorolac to five days [65] and enhance the duration of R-ketorolac administration for peri-operative or post-operative therapy. Studies focused on R-ketorolac would also help resolve the question of whether reported survival benefits are due to the unexpected and potentially fortuitous dual pharmacologic activities in the approved racemic drug. 

## 7. Patents

“Modulators of GTPases and Their Use (NSAIDs)” Inventors: Angela Wandinger-Ness, Larry A. Sklar, Tudor I. Oprea, Laurie Hudson and Zurab Surviladze. United States Patent and Trademark Office award 9,125,899. September 8, 2015.
